# Prevalence and Impact of Simulation Substitution on Paramedic Educational Program Success: A National Examination

**DOI:** 10.7759/cureus.102626

**Published:** 2026-01-30

**Authors:** Hussam E Salhi, Kim McKenna, Christopher B Gage, Jonathan R Powell, Lisa Collard, Michael G Miller, Shea L Van den Bergh, Ashish R Panchal

**Affiliations:** 1 Department of Emergency Medicine, The Ohio State University Medical Center, Columbus, USA; 2 Department of Education, National Registry of Emergency Medical Technicians, Columbus, USA; 3 Department of Emergency Medical Services (EMS) Research, National Registry of Emergency Medical Technicians, Columbus, USA; 4 Committee on Accreditation of Educational Programs for the Emergency Medical Services Professions, Commission on Accreditation of Allied Health Education Programs, Rowlett, USA

**Keywords:** certification, emergency medical services (ems), paramedic education, prehospital, simulation

## Abstract

Objectives

Paramedic educational programs require sufficient rigor to prepare students to perform complex, life-saving skills. Occasionally, live clinical training opportunities are unavailable, leading to simulated skill substitution to meet training requirements. The objective of this study is to describe the prevalence of simulation substitution for clinical skills and its association with paramedic educational program success.

Methods

The current study is a cross-sectional evaluation of paramedic educational programs graduating students in 2019. The study utilizes the Committee on Accreditation of Educational Programs for the Emergency Medical Services Professions' annual report, which includes data on program demographics, outcomes, and questions on the use of simulation. Simulation substitution was assessed using a composite of 11 commonly taught adult/pediatric clinical skills. Descriptive statistics (n, %) and multivariable logistic regression modeling (odds ratio (OR), 95% confidence intervals (CI)) were used to describe the association of simulation substitution frequency and first-attempt certification exam success, defined as a program pass rate ≥75%, adjusting for program characteristics.

Results

A total of 640 paramedic educational programs met the inclusion criteria. Of these, 59% (379/640) had simulation substitution of skills, with the extent of substitution being variable (1-5 skills: 25% (161/640); 6-7 skills: 18% (118/640); ≥8 skills: 16% (100/640)). Paramedic program first-attempt certification examination success decreased as simulation substitution increased above five skills (OR, 95% CI: 1-5 skills: 0.71, 0.46-1.08; 6-7 skills: 0.51, 0.32-0.82; ≥8 skills: 0.53, 0.32-0.87; referent 0 skills).

Conclusions

Simulation substitution for isolated clinical skills is common in paramedic educational programs; however, frequent reliance on simulation may reduce the likelihood of first-attempt program success on the national certification examination.

## Introduction

As advanced life support clinicians who provide evidence-based prehospital care, paramedics are vital to the United States (U.S.) emergency medical services (EMS) workforce. Many patients treated in the prehospital setting present with complicated medical or traumatic conditions requiring considerable breadth of knowledge, clinical judgment, diagnostic capabilities, and procedural skill proficiency to treat and transport effectively [1]. As such, paramedic education must be sufficiently rigorous to produce competent entry-level clinicians equipped with an understanding of both medical knowledge and procedural skills. This process includes completing an accredited paramedic educational program, obtaining national certification, and obtaining state licensure [1,2].

A crucial part of paramedic education is training students to perform procedural skills that require both formative and summative hands-on training to produce competent entry-level clinicians. Optimally, students will be exposed to direct clinical training experiences through clinical rotations (e.g., pre-hospital, emergency department). At times, live clinical training opportunities are unavailable, leading to simulated substitution for required training [3]. Simulation training utilizes educator-controlled techniques or environments to represent authentic EMS events, allowing trainees to acquire knowledge, assess performance, practice skills, and deepen their understanding of systems or human action [4]. EMS educational programs increasingly rely on simulation more than other allied professions, which may be due to the number of high-acuity EMS procedures, training limitations during or after the COVID-19 pandemic, or shorter curricula than other allied health professions [5,6].

The extent of simulation substitution in paramedic educational programs, as well as its association with program outcomes and student performance, remains unclear. The objective of the current study was to describe the prevalence of simulation substitution for procedural skills in paramedic educational programs. Additionally, the association of simulation substitution use with paramedic educational program-level success was evaluated.

This study was previously presented as a meeting abstract at the annual meeting of the National Association of Emergency Medical Services Physicians (NAEMSP) on January 14, 2022. San Diego, CA, USA.

An abstract for this work is published in Prehospital Emergency Care. February 2022, vol. 26, no. 1.

This study was previously presented as a meeting abstract at the Society for Academic Emergency Medicine (SAEM) Midwest annual meeting on September 14, 2023. Grand Rapids, MI, USA.

## Materials and methods

Study design and participants

The current study is a retrospective cross-sectional analysis of the 2019 Committee on Accreditation of Educational Programs for the EMS Professions (CoAEMSP) annual report survey, completed by all accredited paramedic educational programs between January and May 2021 [7]. Included in this study were all paramedic educational programs with graduating students in 2019, with either accreditation from the Commission on Accreditation of Allied Health Education Programs (CAAHEP; Seminole, Florida, US) or a Letter of Review (LOR), issued by CoAEMSP, indicating a program is on the pathway to becoming accredited. This accreditation process establishes a requirement of a 100% response rate for all paramedic educational programs, since failure may result in possible penalties, including fees or administrative probation for not completing it on time. The annual report is collected two years after the completion of the educational year to ensure that all candidates have the requisite two-year national certification testing period. The 2021 CoAEMSP Annual Report collected data for the paramedic educational program graduates in 2019. Programs were excluded if they did not have students graduating in 2019. The American Institutes of Research Institutional Review Board (Arlington, Virginia, USA) determined that evaluations using this deidentified publicly available dataset are exempt (reference number: EX0044).

Every annual report contains a set of standard program demographic questions, previously described, along with a unique set of survey questions that differ by year [7]. Data are self-reported by paramedic educational programs during completion of the annual survey. In this annual survey, the unique set of survey questions evaluated the use of simulation in paramedic educational programs. This dataset is collected, cleaned, and collated by CoAEMSP and converted into a deidentified dataset for analysis.

Outcome

The primary outcome was the paramedic educational program's first-attempt success on the national certifying examination, defined as a program pass rate ≥75% or <75%, as previously described by Margolis et al. [8].

Exposure

The study’s primary exposure was the use of simulation substitution by paramedic educational programs as reported in the annual report.

Measures

Simulation substitution was defined as a skill trained through simulated methods as a replacement for direct patient interaction. Programs indicated how often (never, rarely, sometimes, often, or all the time) simulation was used to replace direct patient interaction for each of the 11 procedures. Programs were grouped by whether programs did (sometimes, often, all the time) or did not (rarely, never) substitute clinical skills with simulation. Programs were categorized by the extent of simulation substitution (0, 1-5, 6-7, ≥8 skills) based on population distribution.

The clinical skills measured included 11 adult/pediatric skills performed during clinical and field internships. These skills were decided a priori by investigators leveraging CoAEMSP’s Student Minimal Competencies, the National Registry of Emergency Medical Technicians Paramedic Psychomotor Competency Portfolio, and experts in paramedic educational training and program evaluation (JRP, MGM, LC, CG, ARP) [9]. These skills included pediatric intubation, intraosseous insertion, electrical therapy, supraglottic airway insertion, childbirth, adult intubation, endotracheal suction, patient assessment (primary or secondary), intravenous access, medication administration, and patient interaction (as defined by the paramedic psychomotor portfolio) [10].

In the CoAEMSP annual report, programs were required to answer questions regarding their program's characteristics. Full-time faculty were categorized into four levels based on the count of full-time faculty associated with a program (0-1, 2, 3, ≥4 faculty). Class size was categorized into quartiles based on student enrollment per cohort (1-11, 12-17, 18-29, and ≥30 students). Total hours of instruction, including all types of paramedic educational hours (didactic, laboratory, clinical, field, and capstone), were categorized into quartiles (<1070, 1070-1174, 1175-1298, >1298 hours). National Association of State EMS Officials (NASEMSO) regions were defined as East, South, Great Lakes, Western Plains, and West. Attrition rates were calculated by dividing the total number of students who did not complete the program by the total number of students enrolled. These rates were further categorized into quartiles (<11%, 11-19%, 20-29%, and ≥30% attrition).

Analysis

Descriptive statistics were calculated for program characteristics and simulation substitution, including means and standard deviations (SD), medians and interquartile ranges (IQR), and/or frequencies and percentages where appropriate.

Multivariable logistic regression modeling (odds ratio (OR), 95% confidence intervals (CI)) was used to describe the impact of simulation substitution on first-attempt national certifying examination program success. Using purposeful selection, measures were chosen for inclusion that may have associations between simulation substitution and program success based on prior work [11,12]. The model adjusted for full-time faculty, class size, NASEMSO region, attrition rate, and hours of instruction. Model fit was assessed using Hosmer-Lemeshow goodness-of-fit testing, with statistical significance set at 0.05. Statistical analysis was performed with the Stata 17 package (StataCorp. 2021. Stata Statistical Software: Release 17. College Station, TX: StataCorp LLC) [13].

## Results

A total of 690 paramedic educational programs (response rate 100%) completed the 2019 CoAEMSP annual report, with 640 programs meeting inclusion criteria (Table 1).

**Table 1:** Paramedic program characteristics for graduating students in 2019 by program pass rate. Abbreviations: IQR: interquartile range; SD: standard deviation; NASEMSO: National Association of State EMS Officials

Characteristic	Programs with pass rate ≥75% (n = 348) n (%)	Programs with pass rate < 75% (n = 292) n (%)	Total (n = 640) n (%)
Total students enrolled (median, IQR)	21 (12–33)	16 (11–27)	18 (12–30)
Graduating programs by NASEMSO Regions			
East	75 (22)	32 (11)	107 (17)
South	116 (33)	138 (47)	254 (40)
Great Lakes	60 (17)	75 (25)	134 (21)
Western Plains	42 (12)	30 (10)	72 (11)
West	54 (16)	18 (6)	72 (11)
Months to completion (median, IQR)	12 (12–16)	12 (12–16)	12 (12–16)
Total hours of instruction (median, IQR)	1200 (1090–1312)	1142 (1035–1283)	1175 (1071–1298)
Total hours of clinical experience	216 (172–280)	216 (168–272)	216 (172–276)
Total hours of field experience	164 (88–241)	160 (90–240)	160 (90–140)
Total hours of capstone field internship	192 (108–253)	144 (89–240)	168 (100–250)
Full-time faculty, n (%)			
0–1	113 (32)	108 (37)	221 (35)
2	103 (30)	92 (32)	195 (30)
3	55 (16)	42 (14)	97 (15)
4+	77 (22)	50 (17)	127 (20)
Conferring degree/credentials awarded, n (%)			
Certificate	306 (88)	260 (89)	566 (88)
Associate's degree	181 (48)	154 (53)	335 (52)
Bachelor degree	12 (3)	1 (0)	13 (2)
Attrition Rate			
Less than 11%	96 (28)	64 (22)	160 (25)
11–19%	81 (23)	76 (26)	157 (25)
20–29%	91 (26)	79 (27)	170 (27)
≥30%	80 (23)	73 (25)	153 (24)
First Attempt Certifying Examination Percentage Pass Rate, mean ± SD	89 ± 9	54 ± 18	73 ± 22

Over half (54%) of the programs (348/640) reported a first-attempt certifying exam pass rate ≥ 75%. Programs had a median of 18 students (IQR 12-30) and were primarily in the South NASEMSO Region. There were 419 (66%) programs that reported having >1 full-time faculty member. Months to completion was a median of 12 months with a median total of 1175 hours of instruction. Almost half (49.5%) of the programs (317/640) reported an attrition rate of <20%.

Available simulation modalities for paramedic educational programs are noted in Table 2.

**Table 2:** Simulation training details for programs graduating students in 2019, by program pass rate. Abbreviation: IQR, interquartile range

Characteristic	Programs with pass rate ≥75% (n = 348) n (%)	Programs with pass rate <75% (n= 292) n (%)	Total (n = 640) n (%)
Simulation modalities available, n (%)			
Task trainer	348 (100)	291 (99)	639 (99)
Simple manikin	345 (99)	292 (100)	637 (99)
Intermediate manikin	323 (93)	274 (94)	597 (93)
Advanced manikin	299 (86)	229 (78)	528 (83)
Virtual reality	35 (10)	32 (11)	67 (10)
Simulation Substitution (median, IQR)	1.5 (0, 6)	4 (0, 7)	3 (0,7)
Simulation Substitution (categorical)			
0	162 (47)	99 (34)	261 (41)
1–5	89 (26)	72 (25)	161 (25)
6–7	54 (16)	64 (22)	118 (18)
≥8	43 (12)	57 (20)	100 (16)

Most programs reported having access to task trainers and simple, intermediate, and advanced manikins. Approximately 10% had access to virtual reality training. Simulation was substituted for the 11 identified skills at a median rate of 3 for the total population, with 59% (379/640) reporting substitution of at least one clinical skill. Simulation substitution was used in ≥8 skills by 100 (16%) paramedic educational programs (Table 2). The distribution of simulation substitution per skill for programs with first-attempt certifying exam pass rates ≥75% and those <75% are noted in Table 3.

**Table 3:** Frequency of simulation substitution per procedure category.

Procedure	Program pass rates ≥75% (n = 348) n (%)	Program pass rates <75% (n = 292) n (%)
Peds intubation	174 (55)	182 (62)
IO insertion	145 (42)	168 (58)
Electrical therapy	139 (40)	150 (51)
Supraglottic airway	121 (25)	139 (48)
Childbirth	137 (39)	146 (50)
Adult intubation	112 (32)	125 (43)
Endotracheal suction	111 (32)	132 (42)
Patient assessment	37 (11)	55 (19)
IV access	25 (7)	46 (16)
Med administration	38 (11)	51 (17)
Patient interaction	33 (9)	54 (18)

Simulation substitution, when evaluated in each skill, was higher in programs with pass rates <75% (Table 3).

In multivariable modeling, the odds of first-attempt certification success decreased as the number of simulated skills increased beyond five. Compared to programs with no simulation substitution, the odds ratios (95% CI) were 0.71 (0.46-1.08) for 1-5 skills, 0.51 (0.32-0.82) for 6-7 skills, and 0.53 (0.32-0.87) for 8-11 skills (Table 4). These data demonstrate a lower likelihood of program-level certification examination success associated with programs simulating >5 skills compared to no simulation substitution.

**Table 4:** Association between high first-time certifying exam success and odds of reporting paramedic educational program simulation substitution using multivariable logistic regression models. *Adjusted for total hours of instruction, full-time faculty, class size, NASEMSO region, and attrition rate. ^†^Hosmer-Lemeshow goodness-of-fit p-values = 0.6077. Abbreviation: CI: confidence interval

Factor	Odds ratio	95% CI	P-value
Clinical Skills Simulated^*†^			
0	Referent	Referent	-
1–5	0.71	0.46–1.08	0.110
6–7	0.51	0.32–0.82	0.006
≥8	0.53	0.32–0.87	0.013

## Discussion

The current study investigated the prevalence of simulation substitution in paramedic educational programs and its association with first-attempt national certification examination success. Greater than 60% of programs utilize simulation substitution for at least one skill, with 21% of programs simulating all 11 skills during paramedic educational training. Substituting simulation in 1 to 5 skill areas did not have an association with program first-attempt pass success rates, while simulation substitution in more than 5 skill areas is associated with lower first-attempt pass rates on national certification examinations. Additionally, programs with first-attempt pass success rates less than 75% demonstrate higher simulation substitution in each of the 11 skill areas compared with programs with success ≥ 75% (Table 3). Although the current use of simulation as a substitute for clinical skills is common in paramedic educational programs, caution must be taken if used too frequently, as increased simulation may be associated with the extent of program certification examination success.

These results differ from the literature in the nursing profession, which supports using simulation substitution in nursing education. This discrepancy could reflect the level of simulation quality within the EMS education landscape. In the National Council of State Boards of Nursing’s (NCSBN) National Simulation Study, a randomized controlled trial, the effects of "high quality" simulation substitution for traditional clinical hours were evaluated [14]. High-quality simulation substitution included a realistic environment (e.g., advanced mannequins, virtual reality) and faculty formally trained in simulation pedagogy on nursing education outcomes. When 50% of traditional clinical hours were replaced by simulation, there was no impact on clinical competency, knowledge assessments, or NCLEX® pass rates [14]. Leveraging this work, the NCSBN established a high-fidelity simulation framework with guidelines to standardize simulation use during prelicensure nursing programs [15,16].

The underlying potential mechanisms behind lower outcomes associated with simulation substitution in EMS are likely multifactorial. These include the reduced exposure to the clinical environment and limited development of decision-making in real-world conditions [3,17]. There are limitations on the quality of simulation and faculty development in a number of programs as well. Consistent with the current study, previous work demonstrated that although 91% of programs report access to an advanced simulation mannequin, only 71% use it in the educational environment, and even less use is reported for higher fidelity simulators [18]. Moreover, a significant proportion reported that the EMS program's physical facilities, simulation equipment, personnel, and faculty development were inadequate [9,18]. Inadequate faculty development was exacerbated by the finding that more than half of programs lack personnel resources to support simulation above minimum faculty hours [19]. Ultimately, this leads to variability in simulation pedagogy. This suggests inconsistency in EMS simulations that may affect their efficacy in training, underscoring the need to evaluate the quality of EMS simulations in the US.

Data evaluating measures of quality EMS educational programs and their associated program success is limited. Educators and accrediting agencies often utilize first-attempt certification pass rates on the National Registry of Emergency Medical Technicians (National Registry) examination as a proxy for program success [20]. More recently, studies have integrated other program variables, including attrition rates and EMS educational program characteristics, to better estimate success [8,12,21,22]. To set goals for educational programs, program success benchmarks have been identified. High-performing programs have previously been described as programs with a first-attempt pass rate ≥75% compared to low-performing programs having a first-attempt pass rate <75% [8]. To achieve competency in national paramedic education, continued rigorous evaluations of program success are needed that account for the impact of program characteristics on student success. In the context of these evaluations, this work provides increasing evidence that EMS educational program success is influenced by not only program-level characteristics but also by the choices made regarding educational delivery.

Limitations

There are several limitations to this study. Since the dataset is CoAEMSP’s annual report, this dataset includes no observations from non-accredited paramedic educational programs. However, since completion of training in an accredited program is required for paramedic national certification, this represents a rare population. Additionally, though programs have access to or utilize various simulation modalities, the current study does not evaluate the quality of simulation provided nor the institutional support for these initiatives. Further, although the prevalence of different modalities of simulation in paramedic educational programs is measured in this study, the current data does not provide enough granularity to determine the level of fidelity of simulations. Finally, the skills assessed for simulation substitution were based on the researchers' a priori decision supported by literature and subject matter expertise [10]. It is possible that the inclusion of other clinical skills could impact results. However, this is somewhat mitigated since 11 skills regularly simulated in paramedic educational programs were chosen and identified using CoAEMSP’s Student Minimum Competencies and the National Registry’s Portfolio.

## Conclusions

Simulation substitution for isolated clinical skills is common in paramedic educational programs. In the current study, simulation substitution in more than five skill areas is associated with lower first-attempt pass rates on national certifying examinations. If used too frequently, simulation substitution may be associated with lower odds of program first-attempt national certification success. As paramedic educational programs continue to use simulation to substitute clinical encounters, it is important to emphasize high-quality and reproducible methods of clinician education.
